# Supplementary material for: Using a physics-informed neural network and fault zone acoustic monitoring to predict lab earthquakes
Source: Nat Commun. 2023 Jun 21;14:3693. doi: 10.1038/s41467-023-39377-6 (PMC10284922; doi:10.1038/s41467-023-39377-6)
Supplement: Supplementary file 1 — Supplementary Information [file 41467_2023_39377_MOESM1_ESM.pdf]

Supporting Information for “Using a physics-informed neural network and fault zone acoustic monitoring to predict lab earthquakes”

Prabhav Borate, Jacques Riviere, Chris Marone, Ankur Mali, Daniel Kifer, and Parisa Shokouhi

[1,2,6] Department of Engineering Science and Mechanics, The Pennsylvania State University, University Park, 16802, USA

[3] Dipartimento di Scienze della Terra, La Sapienza Universit`a di Roma, Roma, Italy

[3] Department of Geosciences, The Pennsylvania State University, University Park, 16802, USA

[4] Department of Computer Science and Engineering, University of South Florida, Tampa, 33620, USA

[5] Department of Computer Science and Engineering, The Pennsylvania State University, University Park, 16802, USA

Content of this file

1. Figure S1: “Stick-slip cycle recurrence interval histogram comparison between p5270 and p5271 experiment dataset”.
2. Figure S2: “Shear stress and slip rate cycles comparison between p5270 and p5271 experiment dataset”.
3. Table S1: “A comparison between the training time of Reference, PINN #1, and PINN #2 models for p5270 experiment.”
4. Table S2: “RMSE comparison between the standalone and TL models for experiment p5271.”
5. Table S3: “A comparison between the training time of standalone and TL models for p5271 experiment.”
6. Table S4: “p5271 experiment constants and material properties learned using PINN #1 & PINN #2 frameworks”.

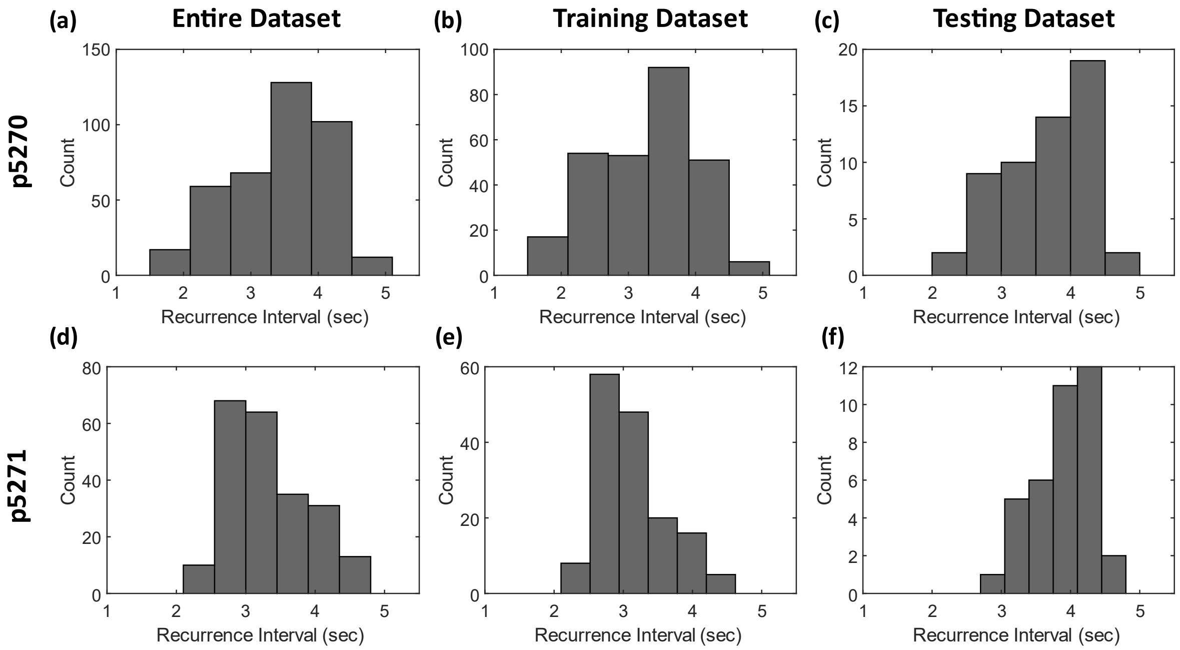

Figure S1: Histograms showing the stick-slip cycles recurrence interval in experiments p5270 and p5271. Plots **(a)-(c)** show the distribution of the cycles in the p5270 experiment for the entire, training (70% data), and testing (20% data) datasets respectively. Similarly, in plots **(d)-(f)** the distribution for the p5271 is shown. The dataset distribution is different for both experiments as acrylic springs of different sizes are used.

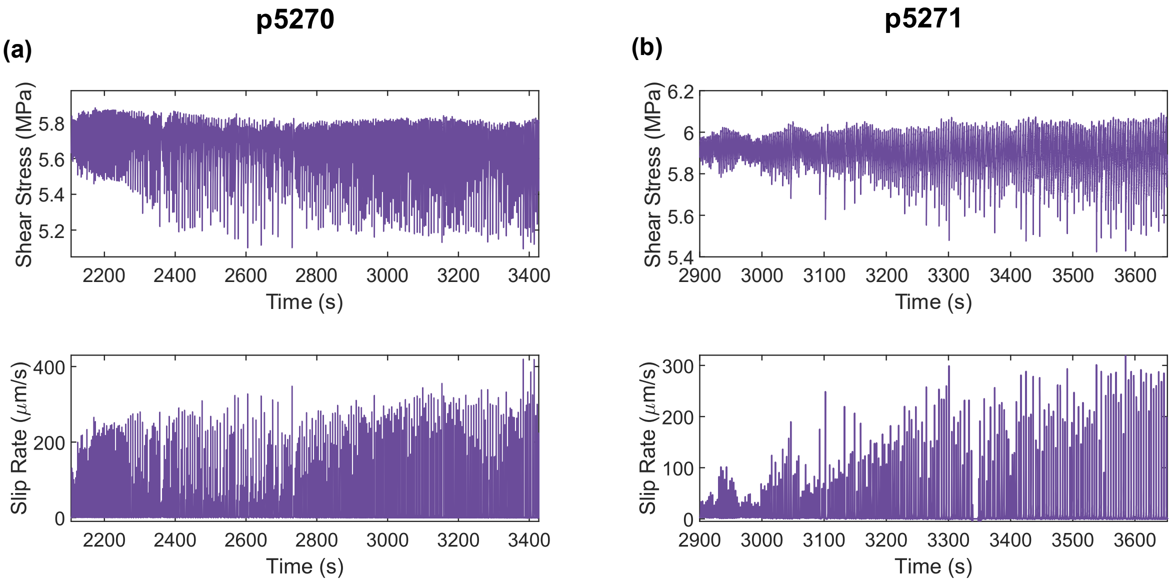

Figure S2: Shear stress and slip rate cycles for both experiments are shown. **(a)** In p5270 the cycles become larger at the beginning of the experiment. **(b)** On the contrary, in p5271 they become larger much later in the experiment (~ 3300 sec).

Model #	Train-Val-Test (%)	Training Time (Sec)		
		Reference	PINN #1	PINN #2
1	70-10-20	619.03	537.47	571.33
2	60-10-20	601.57	501.32	475.00
3	50-10-20	638.91	595.05	448.33
4	40-10-20	451.43	331.17	382.33
5	30-10-20	383.02	262.26	263.67
6	20-10-20	262.33	214.26	258.67
7	10-10-20	167.57	138.68	165.67
8	5-10-20	112.16	107.83	106.33

Table S1: A comparison between the training time of Reference, PINN #1, and PINN #2 models for p5270 experiment.

Model #	Train-Val-Test (%)	RMSE			
		Standalone	TL: Data-driven	TL: PINN #1	TL: PINN #2
1	70-10-20	0.148	0.125	0.095	0.129
2	50-10-20	0.160	0.138	0.114	0.137
3	30-10-20	0.149	0.128	0.124	0.133
4	10-10-20	0.207	0.176	0.132	0.156

Table S2: RMSE comparison between the standalone and TL models for experiment p5271.

Model #	Train-Val-Test (%)	Training Time (Sec)			
		Standalone	TL: Data-driven	TL: PINN #1	TL: PINN #2
1	70-10-20	356	347	341	257
2	50-10-20	282	238	226	227
3	30-10-20	166	153	138	124
4	10-10-20	128	115	110	119

Table S3: A comparison between the training time of standalone and TL models for p5271 experiment.

Table S4 Text:

The experiment constants learned by TL PINN #1 and TL PINN #2 frameworks are compared with their true values in the following Table S4. The parameters k, K, v_l, and ρ are estimated with errors ≤ 10 %, whereas the σ shows more than 15 % error. The constants learned by both models show little to no change across all considered data splits. The reason behind this observation might be the pre-trained weights of the best p5270 models. To initialize the TL PINN #1 and TL PINN #2 models, the best performing p5270 PINN #1 and PINN #2 models are used, respectively. As all the TL PINN models (with varying training data %) start with the same initialization, they may be converging and result in similar trained weights at the end of the training.

Model #	Train-Val-Test (%)	σ (MPa)	k (MPa/μm)	v_l(μm/s)
1	70-10-20	11.84 (+19%)	0.0112 (-7%)	8.30 (-7%)
2	50-10-20	11.85 (+19%)	0.0112 (-7%)	8.31 (-7%)
3	30-10-20	11.85 (+19%)	0.0112 (-7%)	8.31 (-7%)
4	10-10-20	11.84 (+19%)	0.0112 (-7%)	8.30 (-7%)
Actual experiment constants		10 ± 0.01	0.0120	8.9

(a)

Model #	Train-Val-Test (%)	σ (MPa)	K (MPa/μm)	v_l(μm/s)	ρ (kg/m^3)	A_{Intact} (a.u.)
1	70-10-20	11.61 (+16%)	0.045 (-10%)	8.0 (-10%)	2381 (-8%)	20277
2	50-10-20	11.58 (+16%)	0.045 (-10%)	8.0 (-10%)	2381 (-8%)	20277
3	30-10-20	11.53 (+15%)	0.045 (-10%)	8.0 (-10%)	2381 (-8%)	20277
4	10-10-20	11.50 (+15%)	0.045 (-10%)	8.0 (-10%)	2381 (-8%)	20277
Actual experiment constants		10 ± 0.01	0.050	8.9	2600	N/A

(b)

Table S4: Experimental constants and material properties pertaining to p5271 experiment learned by: **(a)** PINN #1 models **(b)** PINN #2 models. The numbers in parentheses show the percentage deviation of the learned constants from the known corresponding values.
